# Supplementary material for: The Proteasome Lid Triggers COP9 Signalosome Activity during the Transition of Saccharomyces cerevisiae Cells into Quiescence
Source: Biomolecules. 2019 Sep 4;9(9):449. doi: 10.3390/biom9090449 (PMC6770237; doi:10.3390/biom9090449)
Supplement: Supplementary file 1 [file biomolecules-09-00449-s001.pdf]

Supplementary Materials

The proteasome lid triggers COP9 signalosome activity during the transition of *S. cerevisiae* cells into quiescence

Laylan Bramasole[1,2], Abhishek Sinha[1], Dana Harshuk[1,2], Angela Cirigliano[3], Sylvia Gurevich[4], Zanlin Yu[4], Rinat Lift Carmeli[1], Michael H.Glickman[4], Teresa Rinaldi[3], and Elah Pick[1,2¶]

[1]Department of Biology and Environment, University of Haifa at Oranim, Tivon 36006, Israel.
[2]Department of Human Biology, University of Haifa, Haifa 31905, Israel.
[3]Department of Biology and Biotechnology, University of Rome "La Sapienza", Rome 00185, Italy.
[4]Department of Biology, Technion - Israel Institute of Technology, 32000 Haifa, Israel.

*corresponding author: Elah Pick: elahpic@research.haifa.ac.il

Includes –

Five supplementary figures

Supplementary references

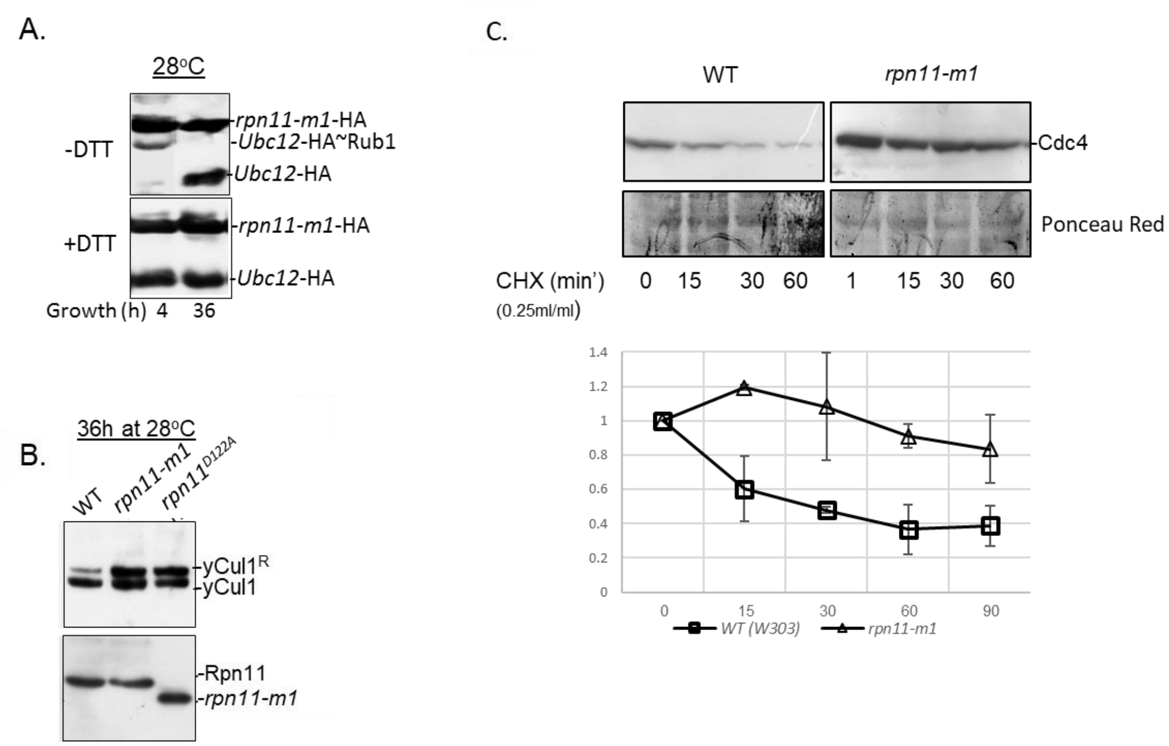

Figure S1 (for figure 1): (A) Overnight grown culture of genomic *rpn11-m1*-HA mutant cells expressing endogenous levels of Ubc12-HA was diluted to OD_{600} = 0.5 in YPD and cultured at a permissive temperature of 28°C for 4 and 36 hours. Ubc12~Rub1 thioester forms were examined in non-reduced (-DTT) and reduced (+DTT) conditions. (B) WT and *rpn11* mutant cells were diluted to $0.5OD_{600}$ in YPD and grown at 28ºC and yCul1 modification status was monitored after 36 hours. Notably, yCul1 status at the post diauxic phase (36 hours) is high in *rpn11* mutants comparing with WT; however, the E2 enzyme (Ubc12-HA) is not forming thioesters with Rub1. (C) WT and *rpn11* mutant cells were diluted to OD_{600} = 0.5 in YPD and grown at 28ºC for 6 hours. After 6 hours cyclohexamide (CHX) was added in a final concentration of 0.25 mg/ml. Equal amount of cells were extracted at indicated times and total protein extraction were used for immunoblotting with anti-Cdc4. Ponceau red staining of nitrocellulose membranes was added to confirm the equal loading of total proteins. Three independent blots were quantified using Image J software and the intensity of Cdc4 is shown (bottom).

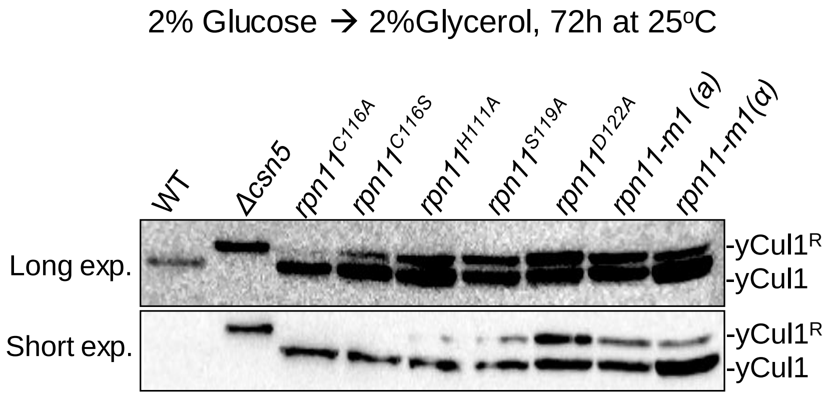

Figure S2 (for figure 1): (A) WT and various *rpn11* mutant strains were grown in YP-Glycerol for 72 hours at 25°C and yCul1 modification status was assessed by immunoblotting. Note that *rpn11-m1* and mutants of the MPN+/JAMM active site (*rpn11*$^{H111/A}$, *rpn11*$^{S119/A}$,and *rpn11*$^{D122/A}$) exhibit a higher status of yCul1^{R} than silent mutations (*rpn11*^{C116A}, *rpn11*^{C116S}) or WT.

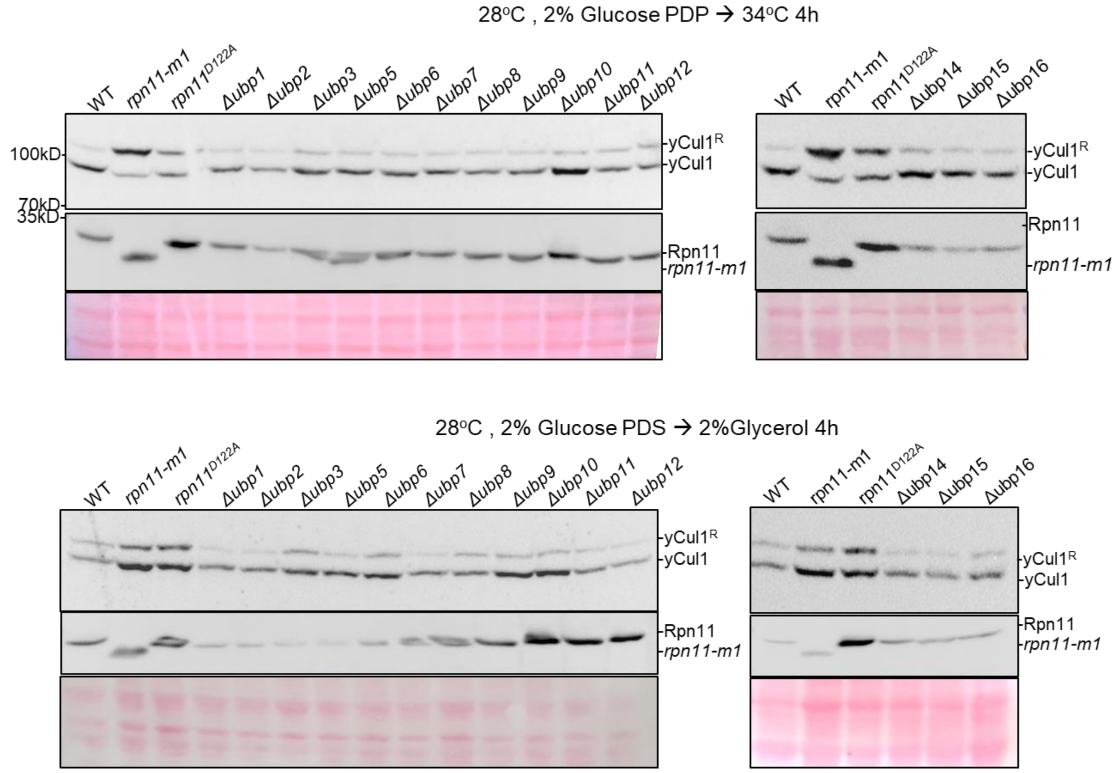

Figure S3 (for figure 1): Various deletion mutants of DUBs were grown in glucose YPD medium until the post-diauxic phase (PDP) followed by four hours at 34°C to induce cell cycle defects (top); or replacement of the growth medium from to YPG medium, containing glycerol as a carbon source. Total cell extracts were used for immunoblotting for yCul1 and Rpn11. Ponceau red staining of nitrocellulose membranes was added to confirm the equal loading of total proteins.

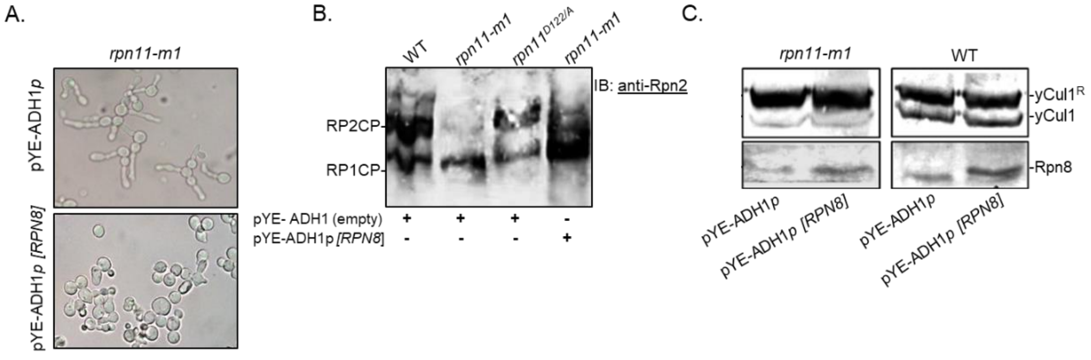

Figure S4 (for figure 2): Suppression of cell cycle and proteasome defects by Rpn8 did not alter yCul1 modification status in *rpn11-m1*. The mutant of *rpn11-m1* has transformed with Rpn8 [*RPN8*] to suppress proteasome and cell cycle defects [1]. Cells were grown overnight at permissive temperature of 28°C followed by additional 8 hours at a restrictive temperature of 37°C to induce cell cycle defects. (A) Suppression of cell cycle defects confirmed by light microscopy (magnification x400, bottom) (B) proteasome structure confirmed by native PAGE, immunoblotted by anti-Rpn2 antibody. The protocol for native PAGE is according to *Yu et al. 2011* [2] . (C) Overexpression of Rpn8 did not alter yCul1 modification status in *rpn11-m1*.

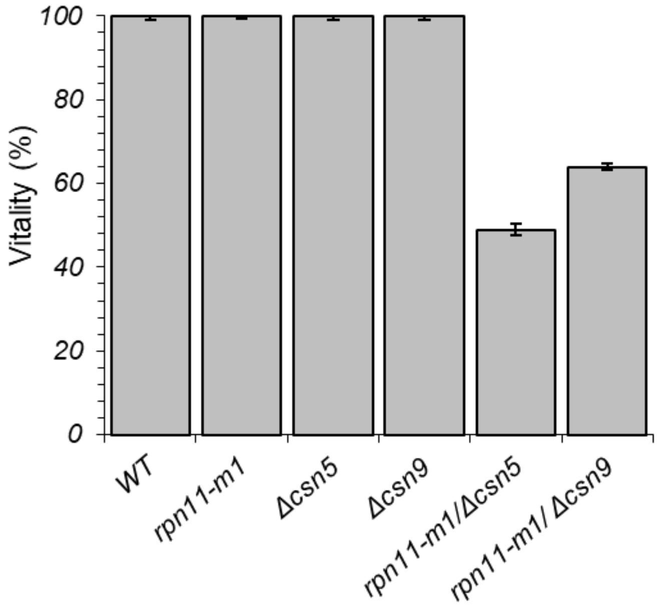

Figure S5 (for figure 5): Vitality test for double mutants of *rpn11-m1* and *csn* components. Two hundred cells of each mutant in the BY4741 genotypic background at logarithmic phase were plated on YPD in triplicates. Agar plates were incubated at 28°C for 2–3 days and the colonies were then counted and percentage of viable cells was calculated. Experiments were repeated three times.

References -

1. Rinaldi, T.; Pick, E.; Gambadoro, A.; Zilli, S.; Maytal-Kivity, V.; Frontali, L.; Glickman, M.H. Participation of the proteasomal lid subunit Rpn11 in mitochondrial morphology and function is mapped to a distinct C-terminal domain *Biochem J.* **2004**, *381*, 275-285.
2. Yu, Z.; Kleifeld, O.; Lande-Atir, A.; Bsoul, M.; Kleiman, M.; Krutauz, D.; Book, A.; Vierstra, R.D.; Hofmann, K.; Reis, N., et al. Dual function of Rpn5 in two PCI complexes, the 26S proteasome and COP9 signalosome. *Mol Biol Cell* **2011**, *22*, 911-920, doi:10.1091/mbc.E10-08-0655.
